# Experimental study on crack propagation pattern and fracture process zone evolution based on far-field displacement by using DIC

**DOI:** 10.1038/s41598-023-44458-z

**Published:** 2023-11-09

**Authors:** Yang Qiao, Xian-bo Guan, Zong-Xian Zhang

**Affiliations:** Oulu Mining School, University of Oulu, Oulu, Finland

**Keywords:** Engineering, Materials science

## Abstract

This study utilizes digital image correlation (DIC) technology to measure the far-field displacements and strains of rock specimens during the entire loading and unloading. Through analyzing the distributions of strain, displacement and their variations per unit length at different stages, the variations of both length and migration velocity of the fracture process zone (FPZ) were studied, and the crack propagation was also investigated. In addition, the entire path of crack propagation was observed by scanning electron microscope (SEM). The results reveal that (1) the fractured ligament can be divided into three zones based on the displacement variation per unit length: intact zone, crack propagation zone, and FPZ. (2) The FPZ length reaches its maximum at the peak load and then decreases, and the minimum length even is only 1/3–1/2 of the maximum length. The FPZ migration velocity is − 48 to 1460 m/s. FPZ’s microscale features are intergranular microcracks, transgranular microcracks, cleavage, and debris on fracture surface and around main crack propagation path. (3) The crack propagation length during peak load to peak-post 90% accounts for more than 1/3–1/4 of the entire post-peak length. Crack propagation is alternating fast and slow, i.e., the velocity of crack propagation varies regularly in the range of 24–700 m/s. The region of crack initial propagation is more severely damaged compared to other propagation regions.

## Introduction

Fracture Process Zone (FPZ) serves as the precursor of the macroscopic crack extension and the window of system energy release, which determines the crack propagation path and affects the value of the system energy dissipation^1–3^. Crack propagation is the main reason for rock instability failure^4^. Rock instability failure is not caused simply by main crack propagation but by the linkup of microcracks in static or quasi-static loading^4^. Therefore, studying the FPZ evolution and crack propagation is of significance in explaining rock instability failure.

The nonlinear zone near crack tip was first proposed in metallic materials by Orowan^5^ to resolve the contradiction between energy release rate G_c_ and surface energy ${\gamma }_{s}$ in Griffith theory^6^. Dugdale and Barenblatt developed a model to describe the internal development mechanism of a damage zone near a crack tip^7,8^. This model was called D–B model and it explained that the damage zone was a region with cohesion and traction force acting on the microscopically separated surfaces surrounding the crack tip. Inspired by the D–B model, Hillerborg et al. proposed the Fictitious Crack Model (FCM) and introduced the concept of the fracture process zone which can reflect strain softening^9^.

Rice et al.^10^ proposed the FPZ’s shielding effect that could reduce the effective stress at the crack tip during crack propagation. Then Knehans and Steinbrech^11^ inferred the two main factors contributing to the shielding effect: crack tip microcracks and grain bridges. Due to the stress concentration at the crack tip, microcracks appear at the weaker region of the material and these microcracks will propagate along the path consuming lower energy. During this process, due to attenuation of the residual stress in the crystal grains as the initiation and propagation of microcracks, the crystal grains originally bound by the matrix begin to expand, which is equivalent to applying compressive stress on the surface of the macroscopic crack, forming the effect of closing the crack. Evans et al.^12^ used the microcrack density and the width of the FPZ to visualize the effect of microcracks. When the proportion of microcracks was not more than 20%, the crack propagation was basically unaffected. When it reached 30%, the fracture toughness would increase by around 10%. The shielding effect mechanism of grain bridging was obtained by Swanson through experiment^13^. By tracking and observing the crack propagation path, it was found that larger grains at the crack tip have the capacity to impede crack propagation. When encountering these larger grains, the crack would change the propagation path, with the larger grains effectively acting as a bridge between the crack surfaces. If the crack continues to propagate, it must overcome the cohesion between the crystal grain and the matrix.

Lin et al.^14,15^ obtained the fractured ligament horizontal displacement of the three-point bending beam at each loading stage by DIC. The FPZ length at the peak load fully developed, and the crack tip opening displacement at the peak load was used as the critical value to measure the FPZ length of the post-peak load. Vavro et al.^16^ studied the FPZ evolution by X-ray real-time scanning imaging technology and determined that the FPZ initiated at 80% peak loading. Unfortunately, due to the sample configuration, the experimental results after the load peak were not available. Based on the linear cohesive zone model, elastic opening displacement and critical opening displacement were also used to identify the three stages of FPZ evolution^17^. Many studies have focused on the evolution of the FPZ during loading^2,16,18^, but few ones involve the effect of unloading (crack propagation) on the FPZ evolution. On the other hand, the initial FPZ size was affected by the notch passivation effect, leading to a deviation between the experiment and the theoretical value of FPZ size. In order to observe the real FPZ size, the volume of the crack is required to be zero, which means that the pre-crack width is zero. Obviously, the sample in the experiment cannot meet this condition. However, the FPZ during the crack propagation will not be affected by the notch passivation effect.

Because the FPZ location is relatively fixed before the peak load, the displacement and strain near the crack tip can be used to analyze the FPZ. As the crack propagates, the FPZ location is constantly changing. In order to accurately monitor the crack and FPZ, the far-field displacement and strain are studied and analyzed in this article. This study focuses on the deformation of the entire fractured ligament during the whole loading and unloading, FPZ evolution and crack propagation pattern.

## Fracture experiment

**Figure 1:**
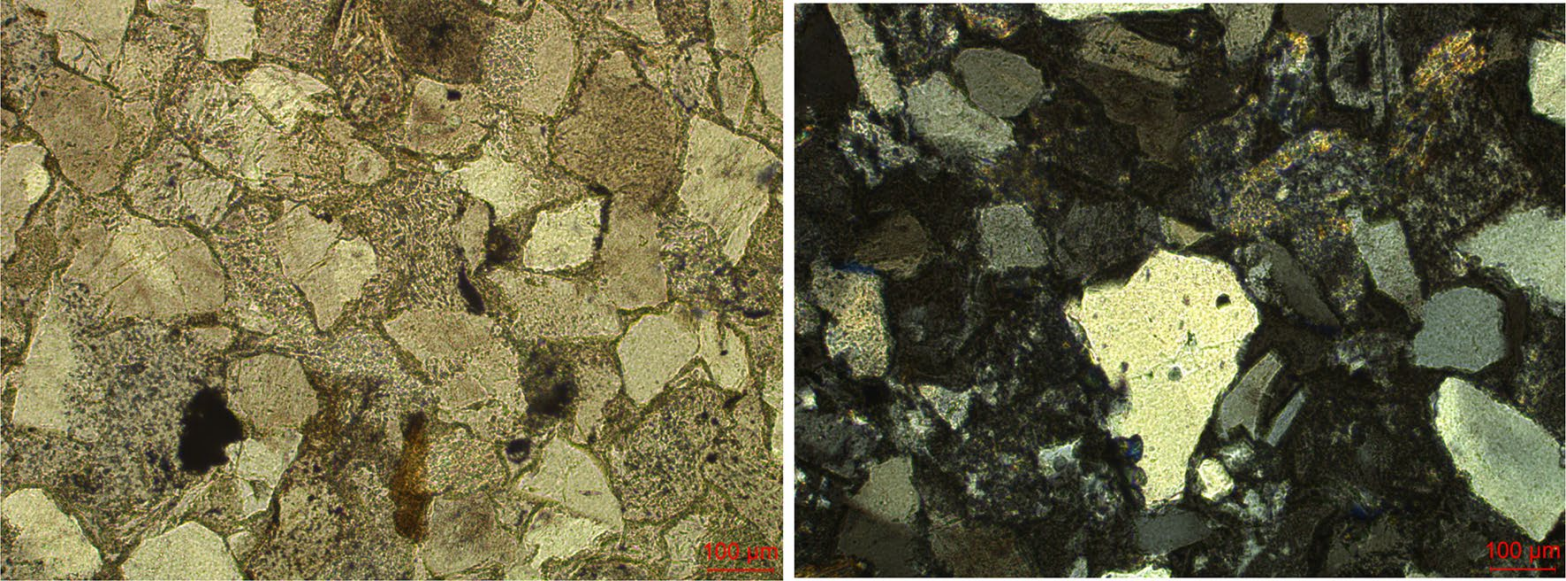
Optical microscopy results, transmitted light (left) and polarized light (right).

A sandstone from a quarry in Sichuan, China was selected to manufacture three-point bending beams. The uniaxial compressive strength is 52.4 MPa^18,19^. Based on the polarizing and transmission micrographs in Fig. 1, the minimum and maximum grain sizes are 0.11 mm and 0.54 mm respectively, and the proportion of the grain sizes between 0.15 and 0.3 mm is about 74%.

**Figure 2:**
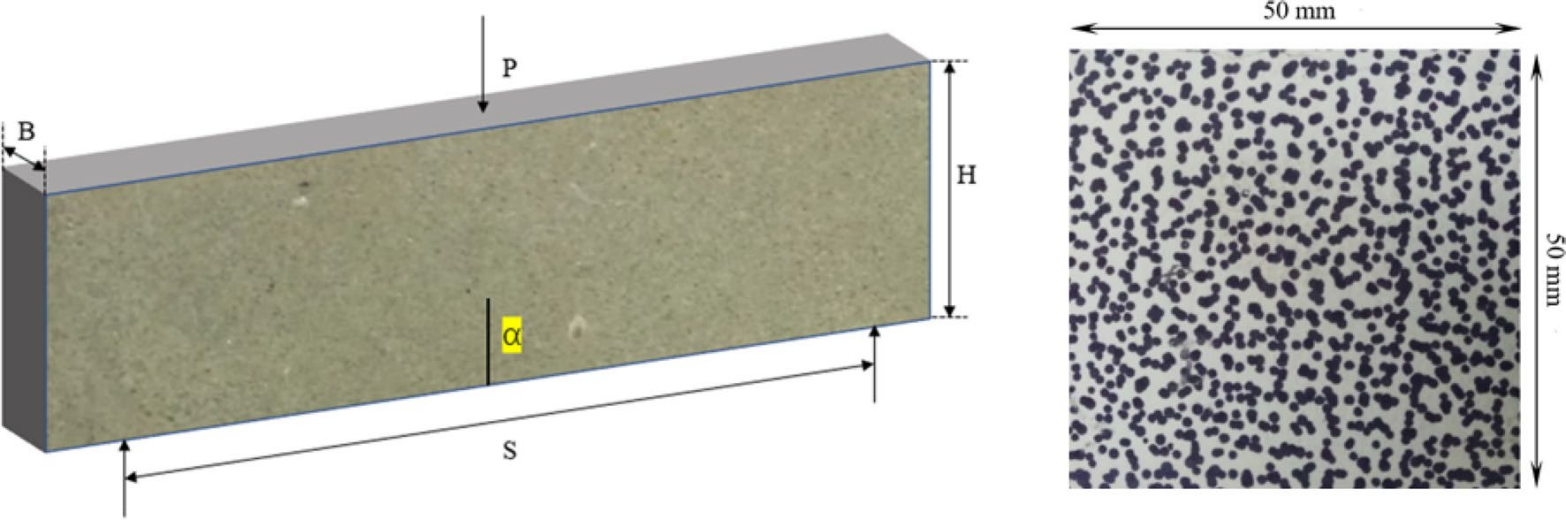
Loading geometry and speckle pattern.

The specimen is three-point bending beam (Fig. 1) with a speckle pattern and a central pre-crack that was created using a diamond wire with a diameter of 0.2 mm, resulting in a pre-crack width of approximately 0.3 mm. The dimensions of the specimens are listed in Table 1. The ideal speckle pattern should have high contrast, non-repetition, and anisotropy, and minimize the gray area. To obtain the surface displacement of specimens, it is necessary to obtain a series of speckle images before and after the deformation in the specimen by a high-speed camera. After the experiment is completed, the displacement of any deformed stage is calculated by using DIC software by comparing with an undeformed speckle image. The undeformed speckle image is generally referred to as Reference Image, and the deformed speckle image is referred to as Target Image. The relative displacement can be calculated using the VIC-2D software of the digital image system. The speckle pattern is shown in Fig. 2.

**Table 1:** Dimensions of specimens.

Specimen no	CNB-1*	CNB-2	CNB-3	CNB-4	CNB-5	CNB-6	CNB-7	CNB-8	CNB-9
Length S (mm)	320	320	320	280	280	280	240	240	240
Height H (mm)	78.9	79.2	79.0	69.9	69.9	70.1	59.7	59.8	59.1
Thickness B (mm)	39.8	39.7	39.7	35.4	35.0	35.3	29.8	29.7	29.8
Pre-crack length α (mm)	23.5	23.1	18	22.2	21.4	23.5	21.4	18.6	17.1
Peak load (N)	464	512	766	360	328	278	184	338	300

B represents the thickness of the specimen, H represents the height of the specimen, and S represents the span length of the specimen during loading. CNB is the abbreviation for Center Notch Beam, the number following CNB represents the number of the specimen.

**Figure 3:**
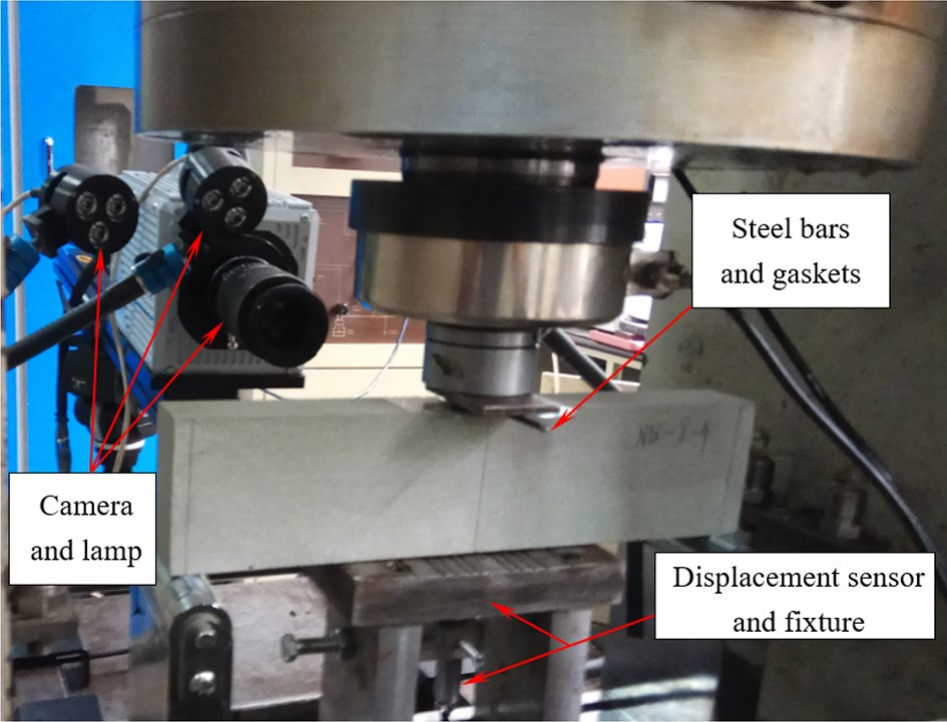
Loading system for the rock specimen.

The experiment utilized a digital imaging system produced by Correlated Solutions Incorporated (CSI), USA, with a CCD (charge-coupled device) camera featuring a maximum effective square resolution of 2048 × 2048 pixels and a 100 mm fixed-focus lens. When the center displacement of the specimen changes each 0.001 mm, a photo is taken. Four LED lights are positioned on both sides of the camera. Considering the susceptibility of optical instruments to contaminants before the loading, it was imperative to ensure that the CCD camera lens was clean. The camera lens was meticulously aligned perpendicular to the test specimen's surface, and the focus was carefully adjusted while positioning the LED light source to ensure uniform illumination of the specimen surface. At the same time, the acquisition software's light intensity histogram was also applied to confirm the adequacy of the lighting arrangement. RMT-150B loading process is controlled by using the beam intermediate deflection as a servo feedback signal, and the loading rate is 0.02 mm/s. Figure 3 shows the specimen loading system, and systematical fractographic studies were performed using a low vacuum SEM.

In this fracture experiment, the DIC was chosen due to its exceptional capacity to provide high-resolution data. DIC observed the deformation behaviors of the rock specimens during the experiment. Throughout the loading and unloading process of the rock specimens, we continuously captured images. These images were then analyzed to track the displacement and strain of the rock surfaces.

## Results and analysis

### Horizontal displacement and strain contour of the crack tip

The collected photos from the experiments are processed by software (VIC-2D) to obtain the horizontal displacement and the horizontal strain. The software sets up coordinates for the observation area automatically, and the subset and step size for processing the speckle photos are chosen based on photo quality and data precision. The subset size is closely related to the data accuracy. The software matches the unloaded subset with the subset after loading to obtain the relevant data point for calculating the displacement of the pixel. A larger subset size provides more relevant points for better matching and displacement accuracy. Therefore, the subset size should be set to as large as possible to improve the calculation accuracy. In this study, the subset is set to 27 pixels. The size of the step is closely related to the data density. The step is the number of pixels between two data points. In this study, the step is 4 pixels, and the average accuracy allowed is set to 0.05 pixels.

**Figure 4:**
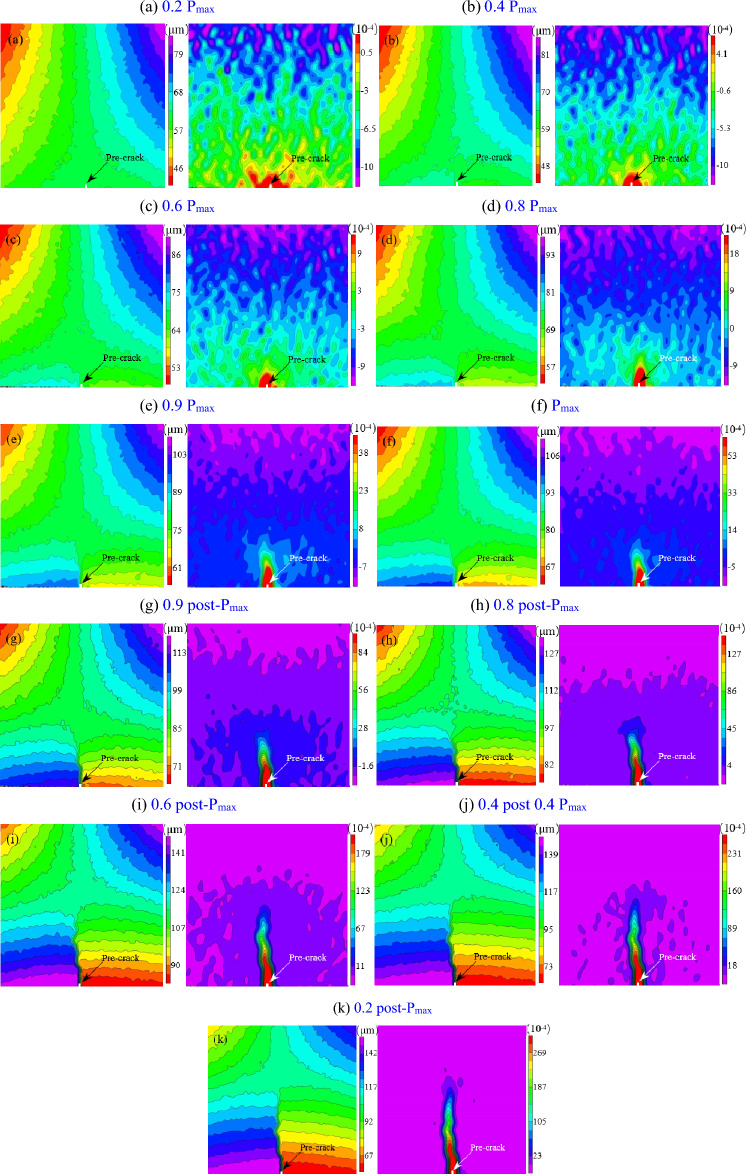
Horizontal displacement (left) and strain (right) result of CNB-3 by DIC.

Figure 4 shows the horizontal displacement and strain during loading and unloading. The horizontal direction is the x-axis and the vertical direction is the y-axis. Each contour line represents the same level of horizontal displacement or strain values with equal differences between adjacent contour lines. Therefore, the density of the contours reflects the variation gradient of the horizontal displacement and strain. The horizontal displacement contours near the crack tip are dense and diverge around the crack tip. The displacement difference on both sides of crack tip is 28 µm for CNB-1, 44 µm for CNB-2, 48 µm for CNB-3, 32 µm for CNB-4, 28 µm for CNB-5, 36 µm for CNB-6, 24 µm for CNB-7, 24 µm for CNB-8 and 31 µm for CNB-9 at the peak. The strain variation within 9 mm in the y-axis direction close to crack tip is 6.0 × 10^–4^ for CNB-1, 5.0 × 10^–3^ for CNB-2, 5.8 × 10^-3^for CNB-3, 7.6 × 10^–4^ for CNB-4, 6.6 × 10^–4^ for CNB-5, 6.4 × 10^–4^ for CNB-6, 5.2 × 10^–4^ for CNB-7, 5.7 × 10^–4^ for CNB-8 and 5.0 × 10^–4^ for CNB-9. The lowest value of the horizontal displacement value suddenly becomes smaller from 90 µm at the post-peak 60% (Fig. 4i) to 73 µm at the post-peak 40% (Fig. 4j), the lowest value continues to decrease at the post-peak 20% (Fig. 4k), and similar phenomena were observed in other specimens as well, where the displacement on one side of the crack tip suddenly decreased after the peak load. These phenomena indicate that the cohesion remaining in the ligament is not enough to support and pull the other side to make displacements decrease at the other side.

### Fracture ligament division

**Figure 5:**
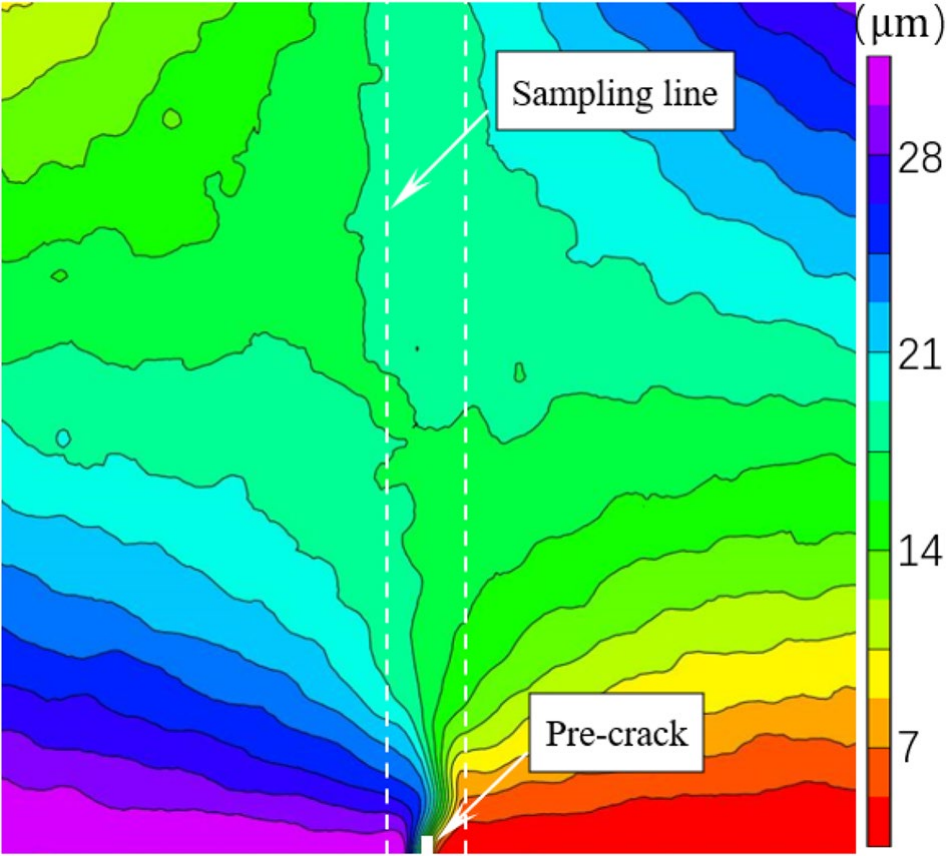
Sampling line distribution of specimen CNB-3.

**Figure 6:**
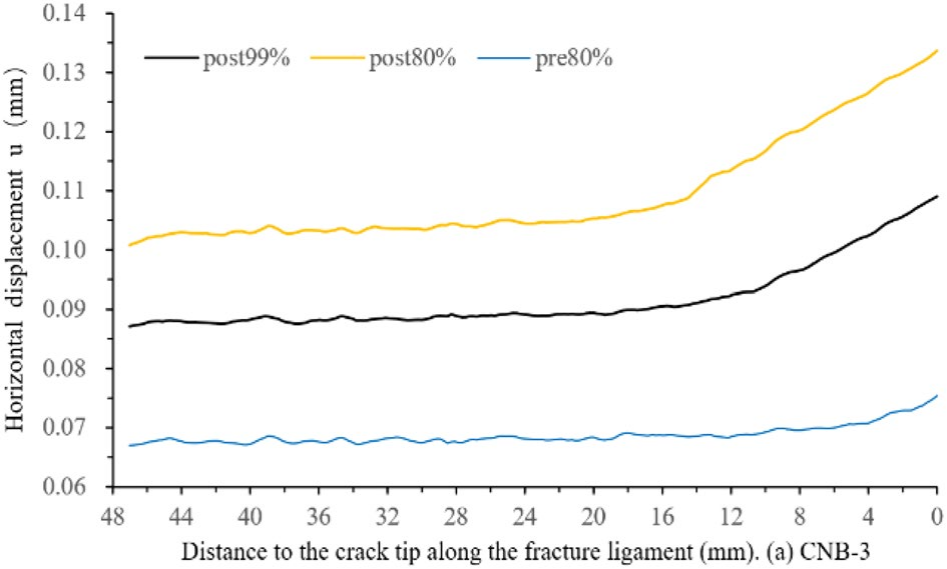
Horizontal displacement along fracture ligament.

**Figure 7:**
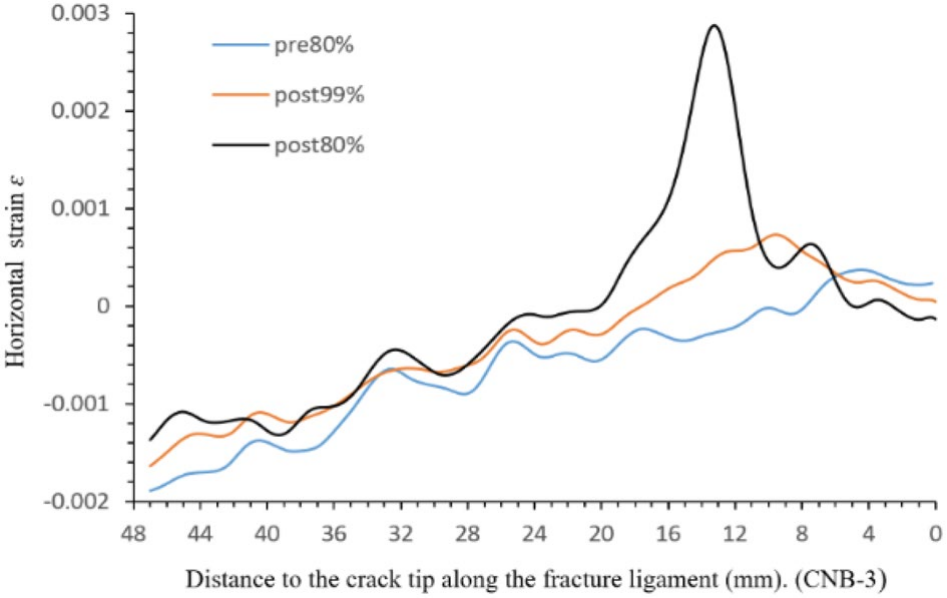
Horizontal strain along fracture ligament.

Fracture ligament is a narrow region between the pre-crack tip and the loading point where the crack propagates and passes through. Based on far-field horizontal displacement along the fracture ligament, MATLAB is used to fit the horizontal displacement function and the best-fit function is determined by comparing its derivative with the horizontal displacement variation per unit length. The distribution characteristics of the derivative (displacement variation per unit length) in the entire fracture ligament are analyzed, then the fracture ligament is divided into three zones, namely the crack propagation zone (CPZ), fracture process zone (FPZ), and intact zone (IZ),

The sampling line is parallel and 2.97 mm away from the pre-crack with a length of 50 mm in Fig. 5. The obtained horizontal displacement values are from pre-peak 80%, peak-post 99% and 80%. The horizontal displacement and strain on the fracture ligament are shown in Figs. 6 and 7. The two figures illustrate the displacement and strain characteristics of the fracture ligament. Combining the derivative value of the horizontal displacement (displacement variation per unit length), the fracture ligament can be divided into three zones: the intact zone (IZ), the fracture process zone (FPZ), and the crack propagation zone (CPZ).

**Figure 8:**
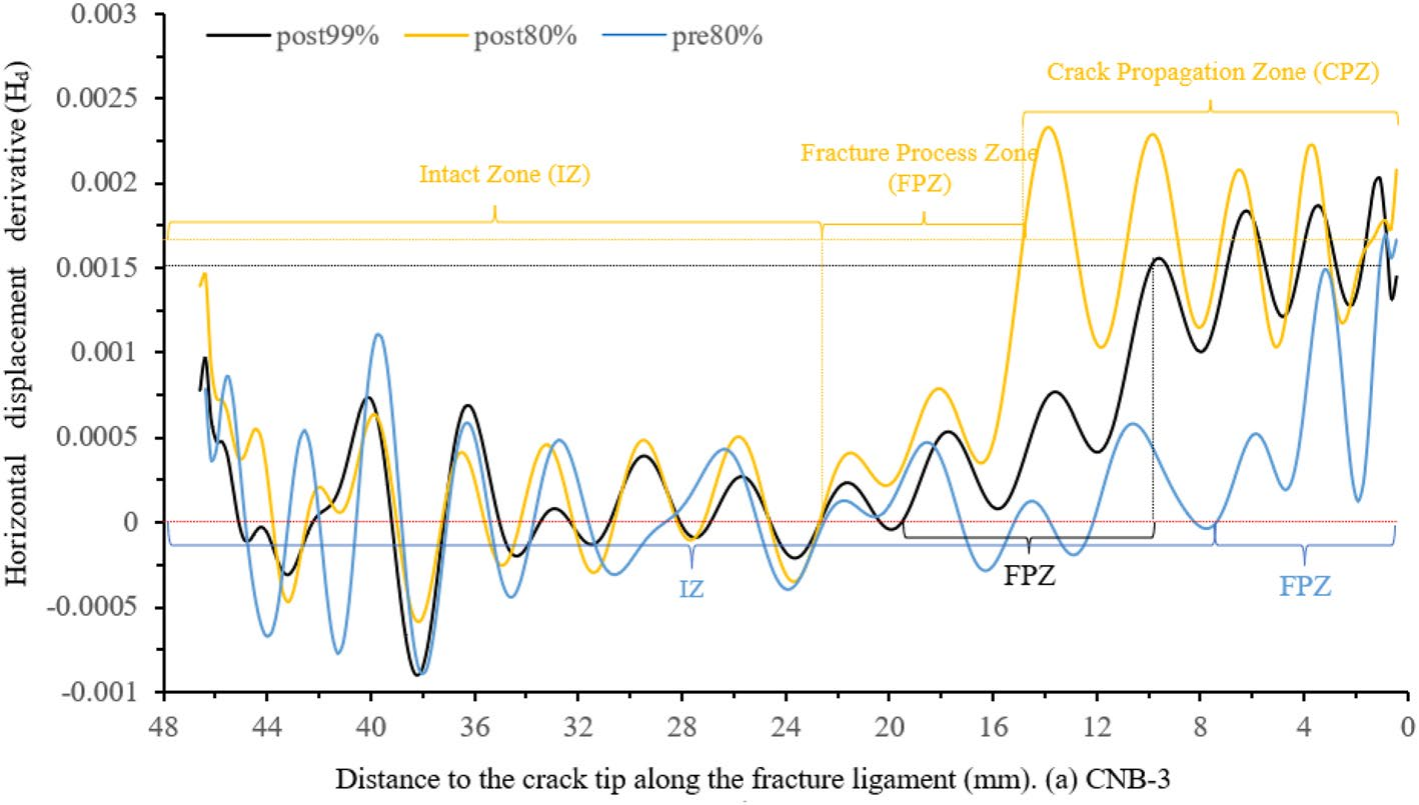
Horizontal displacement derivative (H_d_) along the fracture ligament.

Using MATLAB obtain the derivative value of the horizontal displacement (displacement variation per unit length), as shown in Fig. 8. This figure illustrates the characteristics of displacement variation in three zones of the fracture ligament: IZ, FPZ and CPZ.

Figure 8 shows that the horizontal displacement derivative curve (displacement variation per unit length) at post-peak 80% can be clearly divided into three sections. At 22.5–48 mm away from the crack tip, the derivative value maintains constant fluctuations around *y* = 0, which is the fluctuation baseline. The derivative values of post-80%, post-99%, and pre-80% are not significantly different, and their fluctuation trend remains consistent at 22.5–48 mm. This means that the state of this region has remained stable from pre-peak 80% to post-peak 80%. In Fig. 8, the horizontal displacement values of the region (22.5–48 mm) remain consistent at the same load moment, with pre-peak 80% being 0.067 mm, post-peak 99% being 0.088 mm, and post-peak 80% being 0.103 mm. The derivative and displacement values comprehensively reflect that the region is undamaged from 22.5 to 48 mm and moves as a whole to the right. Therefore, the region where the horizontal displacement derivative value fluctuates with 0 as the fluctuation baseline is defined as the intact zone (IZ).At the post-peak 80%, the horizontal displacement derivative value gradually increases at 14.6–22.5 mm with the curve away from the 0-fluctuation baseline, and the horizontal displacement also shows an increasing trend in Fig. 6. The horizontal displacement and derivative of post-peak 99% also have similar results, which appear at 9.8–19.8 mm. The pre-peak 80% curve rises away from the 0-fluctuation baseline at 7.45 mm and the derivative increases from 7.45 to 0 mm around the crack tip. The strain value not only changes from compression to tension but also has a high peak, as shown in Fig. 7. Considering the deformation characteristics of the FPZ^20–22^, the region corresponding to the rising section on the horizontal displacement derivative curve is defined as the fracture process zone (FPZ).

The derivative curve of post-peak 80% enters a stable fluctuation state at 0–14.6 mm, the fluctuation baseline is y = 0.0017. This indicates that the displacement is linearly increasing. This is also confirmed by the horizontal displacement distribution curve in Fig. 6. The horizontal displacement and derivative at post-peak 99% also appear similar phenomena at 0–9.8 mm, and the fluctuation baseline is *y* = 0.0015. The pre-peak 80% is before the peak and there is no crack propagation, so there is no such phenomenon. Therefore, the region where the horizontal displacement derivative curve stabilizes at a non-zero fluctuation baseline is defined as the crack propagation zone (CPZ).

### Initiation and development of the FPZ

**Figure 9:**
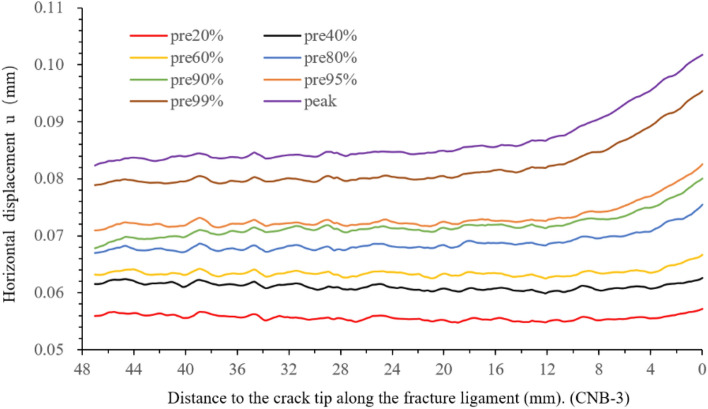
Horizontal displacement versus the distance to the crack tip along the fracture ligament.

**Figure 10:**
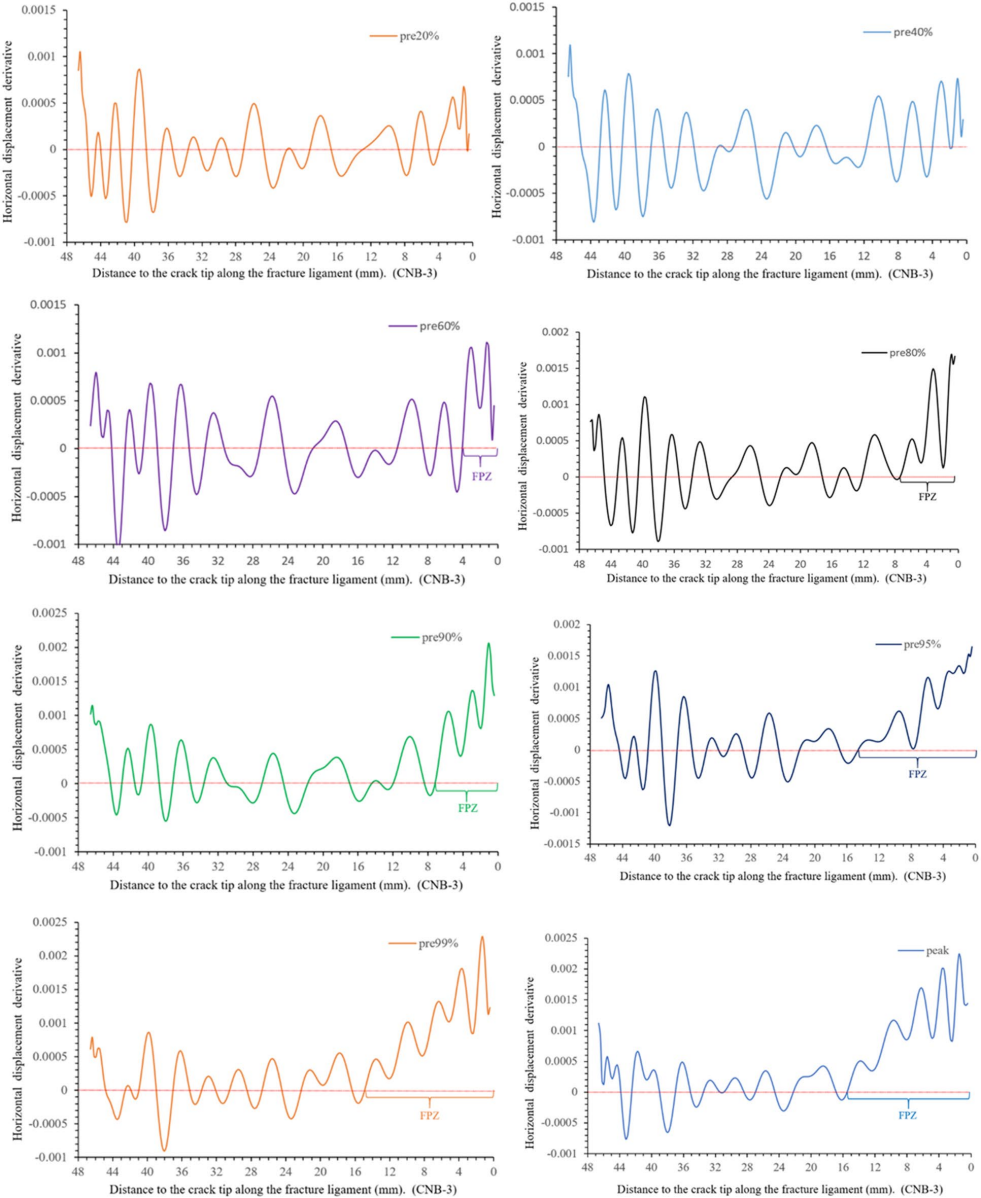
Horizontal displacement derivative versus the distance to the crack tip along the fracture ligament.

To observe the deformation of fractured ligament before the peak load, the horizontal displacement values of the sampling line are extracted at pre-peak 20%, 40%, 60%, 80%, 90%, 95%, and 99%, as shown in Fig. 9. Then obtain the displacement variation per unit length (horizontal displacement derivative), as shown in Fig. 10. The two figures can determine the moment of FPZ initiation and FPZ’s developmental characteristics.

In Fig. 10, displacement variation per unit length (horizontal displacement derivative) remains basically stable at pre-peak 20% and pre-peak 40%. The overall fluctuation range remains consistent, with a range of − 0.0005 to 0.0005 for pre 20% and pre 40%. This indicates that the entire fracture ligament is intact without significant damage. However, it deviates from the 0-fluctuation baseline at 0–4 mm near the crack tip, and the horizontal displacement slightly increases (Fig. 9).

From pre-peak 60% to the peak load, the derivative value near the crack tip increases and deviates from the 0-fluctuation baseline, and the horizontal displacement increases significantly (Fig. 9). According to the FPZ definition in section “Fracture ligament division” and the deformation characteristics of the FPZ initiation and development, it can be inferred that the FPZ clearly appears in the crack tip after the pre-peak 60%. The FPZ length is 4 mm at pre-peak 60%, pre-peak 80% is 7.45 mm, pre-peak 90% is 7.2 mm, pre-peak 95% is 14.5 mm, pre-peak 99% is 14.89 mm, and the peak load is 15.5 mm. The FPZ rapidly develops during the pre-peak 90 to the load peak. Correspondingly, the length of the intact zone gradually decreases. The horizontal displacement derivative fluctuate surrounds the 0-fluctuation baseline in the intact zone, and the fluctuation range is still − 0.0005 to 0.0005. In brief, The FPZ length reaches its maximum at the peak load and then decreases, and the minimum length is only 1/3–1/2 of the maximum length.

In rock material, mechanically induced microdamage is generally classified as intergranular microcracks and transgranular microcracks. Cleavage is a subset of transgranular microcracks but important enough in rocks to be treated separately. In addition, some typical microcrack modes, such as opening microcracks, microbranches, cement fracture and surface debris, are often overlooked. The occurrence of different microcrack types depends on the microcracking mechanism and on the microstructure.

**Figure 11:**
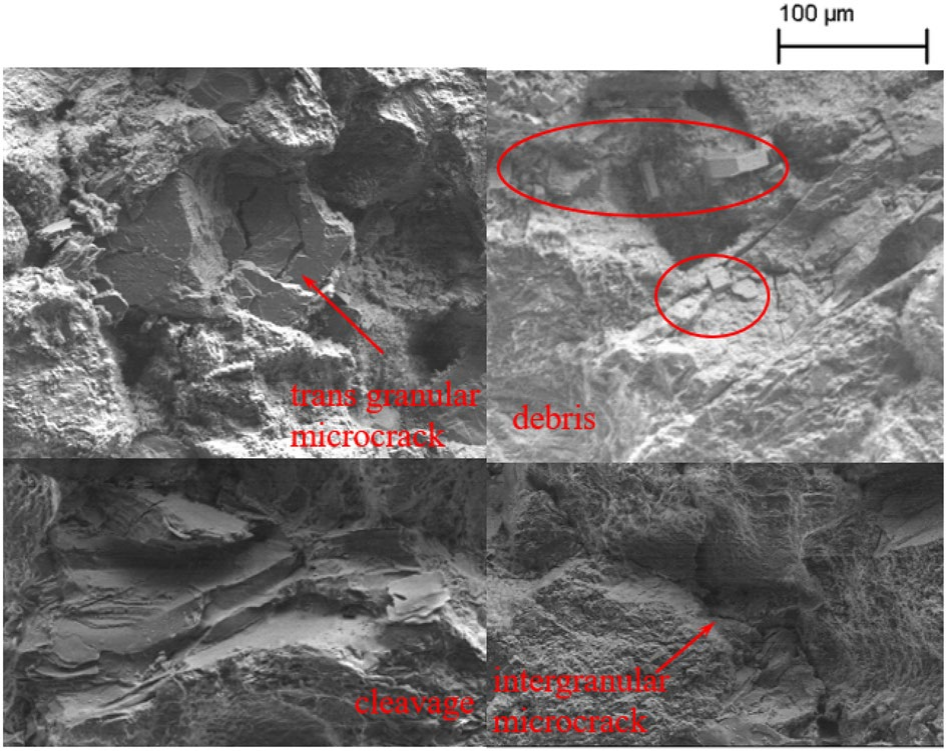
Microdamage on fracture surface.

**Figure 12:**
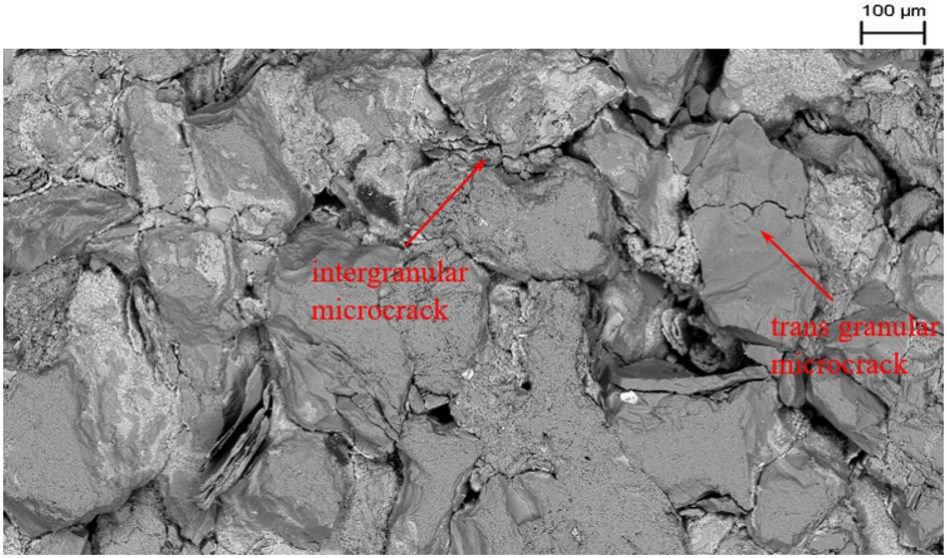
Microdamage in the region around the crack propagation path.

**Figure 13:**
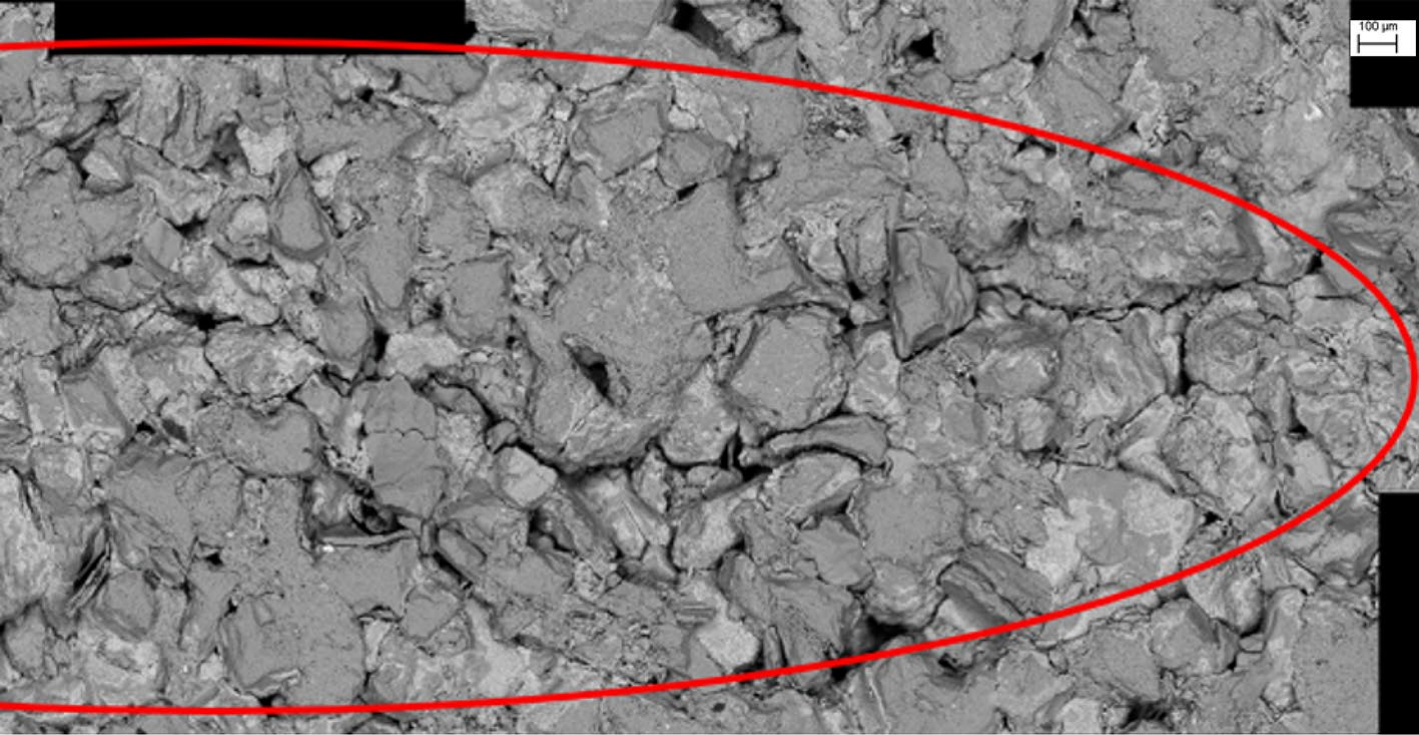
Entire FPZ end (surface) damage.

The entire path of crack propagation and the surrounding region is observed, and the fracture surface is not excluded. Observation of this fracture surface yielded an abundance of FPZ microscale features. Specific regions of intergranular microcracks, transgranular microcracks, cleavage and debris are marked in Fig. 11. Debris on the fracture surface is the main source of fines. A multitude of intergranular and transgranular microcracks are also present in the region around the crack propagation path. As shown in Fig. 12. The entire FPZ end (surface) damage is shown in Fig. 13.

Figures 11, 12 and 13 display the SEM results of the rock specimen, showing the damage characteristics of FPZ and microscopic structures in the fracture surface. Based on the SEM observed result, it is a common trend that the grain boundary is substantially weaker than that of the grain, and hence the microdamage and macroscopic crack appear in grain boundaries in the FPZ.

### Crack propagation and FPZ evolution

**Figure 14:**
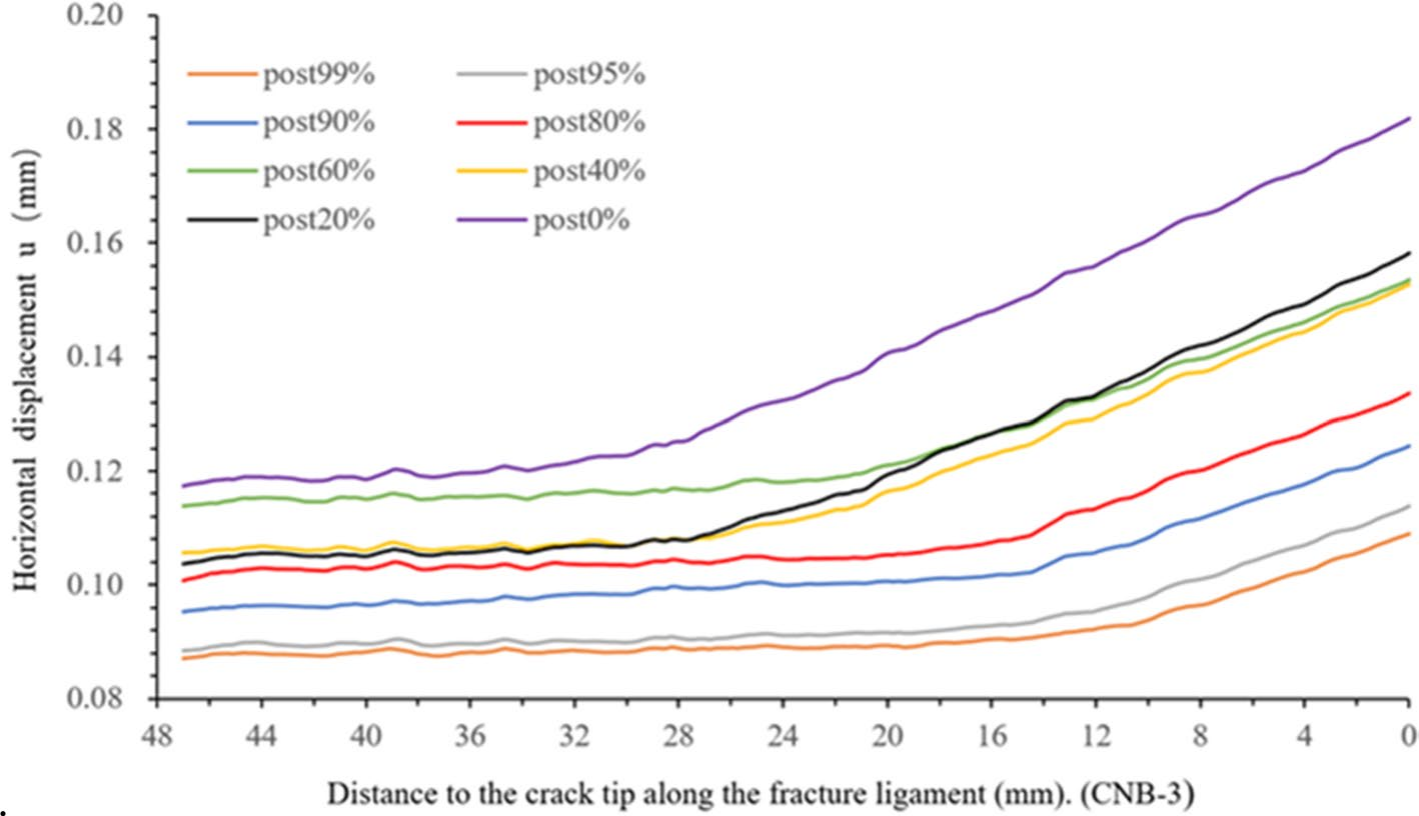
Horizontal displacement versus the distance to the crack tip along the fracture ligament.

**Figure 15:**
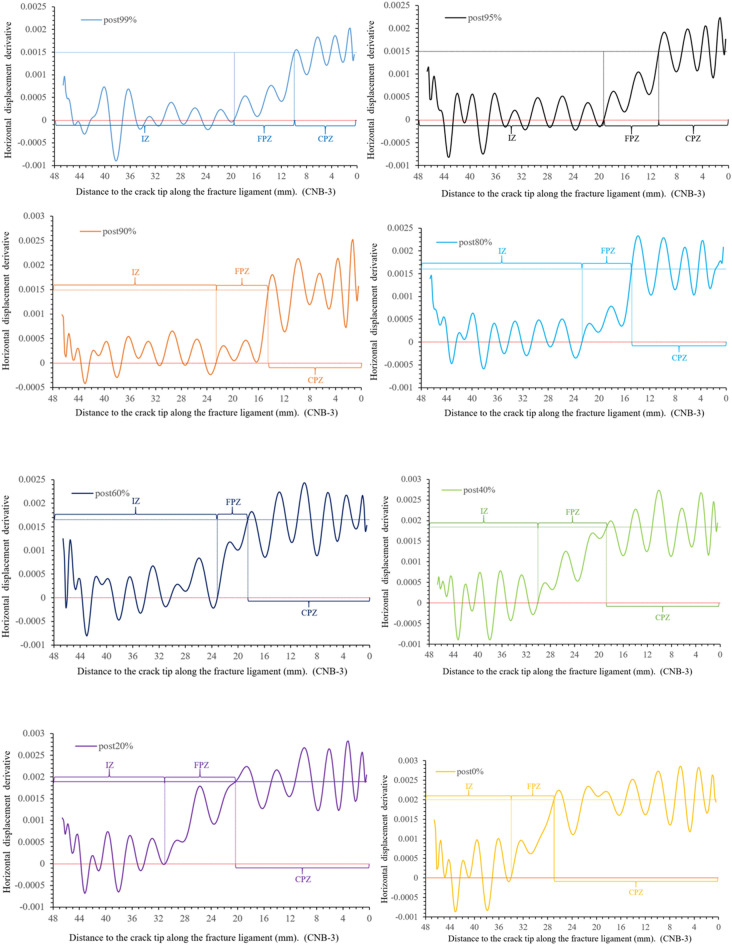
Horizontal displacement derivative versus the distance to the crack tip along the fracture ligament.

This section will focus on whether the FPZ size changes during the crack propagation and how the crack propagation and FPZ migration velocity vary. To investigate the above issue, post-peak horizontal displacement is obtained along the sampling line (post-peak 99%, 95%, 90%, 80%, 60%, 40% and 20%), and the corresponding displacement variation per unit length (horizontal displacement derivative) is calculated. Figures 14 and 15 determine the length of FPZ and the crack propagation zone, and the results are displayed in Table 2.

**Table 2:** Length of the crack propagation zone and FPZ.

Loading moment	Post 99%	Post95%	Post 90%	Post 80%	Post 60%	Post 40%	Post 20%	Post 0%
FPZ length (mm)	9.8	8.56	8	7.9	4.7	11.5	10.3	7
length of CPZ (mm)	9.8	10.8	14.5	14.9	18.5	18.72	20.5	27

On the horizontal displacement derivative curve of post-peak 99% (Fig. 15), it can be observed that the derivative value steadily fluctuates around a baseline of 0.00155 in the region of 0–9.8 mm near the crack tip, while the horizontal displacement shows an almost linear increase (Fig. 14). According to the definition of the crack propagation zone in section “Fracture ligament division”. This region is defined as the crack propagation zone. The length of the crack propagation zone is 9.8 mm at post-peak 99%, and the crack extends to *x* = 9.8 mm on the fracture ligament. The FPZ length is 9.8 mm by measuring the rising section of the derivative curve. At the post-peak 95%, the derivative value near the crack tip is still stable with 0.0015 mm as the fluctuating baseline, but the region has grown to 10.8 mm compared to post 99%. This implies that the crack extends at *x* = 10.8 mm. According to the range of the curve rising, the FPZ length is 8.56 mm and the boundary of FPZ is determined to be x = 19.36 mm. The amplitude of the fluctuation is slightly increased compared to post 99%. According to the above method, the length of the crack propagation zone and FPZ at other loading moments are determined, as shown as Table 2. The crack propagation length during peak load to peak-post 90% accounts for more than 1/3–1/4 of the entire post-peak length.

**Figure 16:**
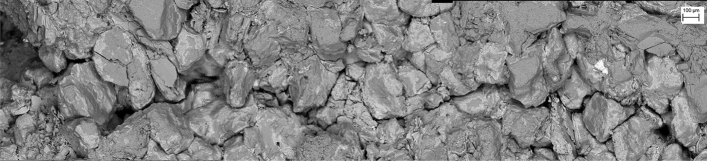
Main crack propagation path and the surrounding region.

The crack propagation path and the surrounding region can be observed by SEM, as shown in Fig. 16. The rock gains around the main crack are loose and many microcracks also appear around the main crack. In the region of crack initiation (or the tip of the pre-crack is on the left side of Fig. 16), the rock material is more severely damaged compared to other regions of the fracture ligament. The direction of crack propagation is vertical to the maximum principal stress.

Figure 16 shows the characters of microscopic structures around crack propagation paths. The region of crack initial propagation is more severely damaged compared to other propagation regions.

According to the length variation of the FPZ in section “Initiation and development of the FPZ” and the FPZ boundaries in Fig. 15, the FPZ migration velocity can be calculated. According to the length variation of the crack propagation zone and the position of the pre-crack tip, the crack propagation velocity can be calculated, and the results are shown in Table 3.

**Table 3:** Velocity of the crack propagation and FPZ migration.

Load moment	Interval (s)	FPZ migration distance L_f_ (mm)	FPZ migration velocity V_f_ (m/s)	Crack propagation distance L_c_ (mm)	Crack Propagation velocity V_c_ (m/s)
Pre60–80%	7.5	3.45	460		
Pre80–90%	7	− 0.25	− 35.7		
Pre90–95%	5	7.3	1460		
Pre95–99%	7.5	0.39	52		
Pre99-peak %	5	0.61	122		
Peak-post99%	5	4.1	820		
Post99–95%	5	− 0.24	− 48	1	200
Post95–90%	5	3.14	628	3.7	740
Post90–80%	5	0.3	60	0.4	80
Post80–60%	9.5	0.4	42	3.6	379
Post60–40%	9	7	777	0.22	24
Post40–20%	13.5	1.6	118.5	1.78	132

It can be found in Table 3 that both the FPZ and the cracks have same velocity pattern during the loading and unloading. The pattern of crack propagation is intermittent, i.e., the velocity of crack propagation varies regularly in the range. During unloading the crack velocity varies largely from 24 to 740 m/s. This is reasonable since crack propagation requires energy. When the energy concentration reaches a critical value (specific fracture energy), the crack will propagate to a certain distance. Then when this energy is used due to the crack propagation, the crack will arrest, or the propagation velocity becomes very small. After that when the next energy concentration reaches a critical value, the crack will propagate again with a high velocity.

## Discussion

In this study, the initiation of FPZ was directly determined by the displacement values, and the initiation of FPZ occurred at 60–70% peak load, which is within the range of 70–90% peak load obtained by Nathan (2018). The length of FPZ is 7–15.5 mm in the study. The FPZ length reaches a maximum (12–15.5 mm) at the peak load. After the peak load, the FPZ length decreases and the minimum length is even only 1/3–1/2 of the peak length. This result is consistent with the previous experiment results by Zhang et al.^18^ who used SCB specimens with diameters of 50 100 150 200 mm to measure the corresponding FPZ lengths with mean values of 4.05, 7.59, 10.62 and 12.87 mm. They also found that the range of the FPZ reaches the maximum at the peak. The closer to the peak, the faster the development of the FPZ, and the length of the FPZ that increases from 90% pre-peak to peak accounts for 50% of the total FPZ length. Many studies have proved that the initiation, development and formation of the FPZ are all at the peak or before^23–26^.

The crack propagation length during the peak-load to peak-post 90% accounts for 1/4–1/3 of the entire post-peak propagation length in this study. The energy stored in the specimen before the peak load is about 1/2 of the total energy. It is used for the FPZ formation and crack propagation during the peak-load to peak-post 90%. When a pre-existing plane crack occurs under external tensile loading, in the energy criterion, crack advance is governed by the strain energy release rate G at the crack-tip^27,28^. Crack nucleation occurs when the energy release rate of the crack-tip is no less than the fracture energy of the matrix^29^. The pattern of crack propagation is alternating fast and slow, i.e., the velocity of crack propagation varies regularly in the range of 24–700 m/s^30^. Measured the crack propagation velocity in the range of 0–2000 m/s. Henry et al.^31^ measured the crack propagation velocity of a micro grained (1–4 μm) limestone and of a fine-grained (100–300 μm) marble in water, and the velocity range is 0.03–1600 m/s. Crack arrest often occurs in crack propagation due to insufficient fracture energy and specimen configuration^32,33^. Regarding crack propagation issues, energy release rate or fracture toughness may explain the above experimental phenomenon. Fracture toughness as a function of crack propagation velocity, in the low velocity range, decreases with increasing crack propagation velocity due to strain rate effects; in the high velocity range, fracture toughness increases with increasing crack propagation velocity due to rising temperatures at the crack tip and a reduction in the degree of multiaxiality of the crack tip stress field^30,34^. During slow propagation of the crack, the fracture energies assume very high values^35,36^.

This study on FPZ is limited to quasi-static loading conditions. It is necessary to investigate FPZ under dynamic loading conditions in which crack branching and layer cracks are found^37,38^. Such crack branching or layer cracks might be one of the sources of fine materials or particles from rock blasting^39–42^. Therefore, it is interesting to investigate the FPZ under dynamic loading conditions in the future.

## Conclusion

According to the distribution characteristics of the derivative value of the far-field horizontal displacement (or displacement variation per unit length), the fractured ligament is divided into three zones. The region where the horizontal displacement derivative takes 0 as the fluctuation baseline is the intact zone, and the region where the horizontal displacement derivative fluctuates around a non-zero value as the fluctuation baseline is the crack propagation zone. The fluctuation baseline of the horizontal displacement derivative is 0.0015–0.002 on crack propagation zone. The region corresponding to the rising section of the horizontal displacement derivative curve is the FPZ.

FPZ microscale features are intergranular microcracks, transgranular microcracks, cleavage and debris on the fracture surface and around the main crack propagation path. The FPZ length reaches a maximum (12–15.5 mm) at the peak load. After the peak load, the FPZ length decreases and the minimum length is even only 1/3–1/2 of the peak length. During the FPZ evolution, the front boundary of the FPZ even would be set back due to microcrack closure.

The crack propagation length during the peak-load to peak-post 90% accounts for 1/4–1/3 of the entire post-peak propagation length. The region of crack initial propagation is more severely damaged compared to other propagation regions; this is the intrinsic mechanism that crack initiation toughness is greater than the propagation toughness. Crack propagation is alternating fast and slow, i.e., the velocity of crack propagation varies regularly in the range of 24–700 m/s.

## Data Availability

The data used to support the findings of the study can be obtained from the corresponding author upon request.
